# Average output polarization dataset for signifying the temperature influence for QCA designed reversible logic circuits

**DOI:** 10.1016/j.dib.2018.05.009

**Published:** 2018-05-09

**Authors:** Md. Abdullah-Al-Shafi, Ali Newaz Bahar, Mohammad Maksudur Rahman Bhuiyan, S.M. Shamim, Kawser Ahmed

**Affiliations:** a Institute of Information Technology (IIT), University of Dhaka, Dhaka, Bangladesh; b Department of Information and Communication Technology, Mawlana Bhashani Science and Technology University, Bangladesh; c University Grants Commission of Bangladesh, Bangladesh

**Keywords:** Quantum-dot cellular automata, Reversible logic circuits, Average output polarization (AOP)

## Abstract

Quantum-dot cellular automata (QCA) as nanotechnology is a pledging contestant that has incredible prospective to substitute complementary metal–oxide–semiconductor (CMOS) because of its superior structures such as intensely high device thickness, minimal power depletion with rapid operation momentum. In this study, the dataset of average output polarization (AOP) for fundamental reversible logic circuits is organized as presented in (Abdullah-Al-Shafi and Bahar, 2017; Bahar et al., 2016; Abdullah-Al-Shafi et al., 2015; Abdullah-Al-Shafi, 2016) [1–4]. QCADesigner version 2.0.3 has been utilized to survey the AOP of reversible circuits at separate temperature point in Kelvin (K) unit.

**Specifications Table**

**Table:** 

Subject area	*Electronics*
More specific subject area	*Nano-electronics*
Type of data	*Table, figure*
How data was acquired	*Data set has been attained with QCADesigner tool*
Data format	*Analyzed*
Data accessibility	*Data is within this article*

**Value of the data**

• Reversible circuits are the essential construction block of reversible logic techniques. This dataset aids researcher to improve the consistency and performance of state-of-the-art digital practices.
• The demonstrated data study can assist the researchers to realize the utmost performing temperature of a distinct QCA circuit.
• The outlined dataset can be utilized to form lossless and resilient communication system and arithmetic logic unit (ALU) in quantum computers.

## 1 Data

This manuscript improve the performance of QCA reversible circuits with separate temperature level. QCADesigner has been applied for all the simulation process. The average output polarization (AOP) for fundamental reversible circuits of Feynman, Double Feynman, Fredkin, Toffoli, Peres, BJN, URG, BVF, MCL, TR, R, NG and SCL gates at separate temperature level is presented in Table 1.

**Table 1:** Average output polarization dataset of reversible logic circuits at separate temperature levels.

Design	Output	Temperature at K														
		1	2	3	4	5	6	7	8	9	10	11	12	13	14	15
Feynman	A	3.484	3.481	3.478	3.470	3.461	3.461	3.454	3.454	3.448	3.446	3.443	3.180	3.104	3.006	2.960
	B	3.384	3.381	3.378	3.372	3.367	3.361	3.357	3.354	3.354	3.348	3.343	3.220	3.110	3.012	2.940
Double Feynman	A	3.516	3.503	3.499	3.498	3.493	3.484	3.466	3.447	3.384	3.310	3.239	3.153	3.112	3.089	3.061
	B	3.510	3.509	3.505	3.499	3.492	3.475	3.438	3.400	3.384	3.330	3.245	3.160	3.114	3.065	3.017
	C	3.510	3.507	3.507	3.505	3.500	3.494	3.470	3.440	3.400	3.380	3.349	3.148	3.098	3.069	3.011
Fredkin	A	3.506	3.500	3.500	3.487	3.481	3.472	3.464	3.443	3.443	3.412	3.339	3.174	3.110	3.054	3.011
	B	3.506	3.500	3.498	3.487	3.478	3.470	3.450	3.332	3.324	3.307	3.295	3.149	3.108	3.078	3.048
	C	3.523	3.517	3.511	3.498	3.487	3.476	3.464	3.458	3.448	3.430	3.418	3.271	3.199	3.108	3.058
Toffoli	A	3.515	3.507	3.503	3.500	3.497	3.490	3.472	3.440	3.390	3.340	3.270	3.178	3.109	3.052	3.008
	B	3.512	3.505	3.500	3.500	3.484	3.468	3.450	3.378	3.340	3.312	3.270	3.179	3.104	3.058	3.012
	C	3.506	3.502	3.501	3.501	3.487	3.460	3.414	3.380	3.330	3.302	3.241	3.155	3.078	3.014	2.968
Peres	A	3.511	3.504	3.489	3.481	3.470	3.449	3.428	3.419	3.406	3.394	3.380	3.180	3.114	3.078	3.018
	B	3.589	3.579	3.568	3.550	3.500	3.448	3.435	3.402	3.392	3.384	3.368	3.140	3.098	3.022	2.978
	C	3.506	3.502	3.502	3.487	3.458	3.447	3.414	3.372	3.332	3.308	3.270	3.125	3.089	3.012	2.971
BJN	A	3.505	3.500	3.495	3.482	3.476	3.461	3.447	3.411	3.391	3.350	3.202	3.178	3.110	3.066	3.005
	B	3.489	3.480	3.471	3.462	3.449	3.424	3.388	3.371	3.338	3.307	3.114	3.078	3.014	3.006	2.968
	C	3.485	3.478	3.451	3.439	3.398	3.369	3.344	3.324	3.296	3.280	3.094	3.013	2.964	2.918	2.872
URG	A	3.505	3.500	3.484	3.460	3.441	3.410	3.376	3.361	3.324	3.207	3.173	3.112	3.076	3.024	2.984
	B	3.505	3.484	3.478	3.461	3.432	3.426	3.356	3.336	3.314	3.169	3.113	3.096	3.044	2.978	2.867
	C	3.477	3.469	3.449	3.431	3.412	3.397	3.372	3.361	3.350	3.158	3.088	3.014	2.954	2.872	2.784
NG	A	3.514	3.511	3.505	3.505	3.492	3.476	3.449	3.390	3.338	3.329	3.270	3.190	3.113	3.087	3.011
	B	3.500	3.496	3.491	3.480	3.468	3.450	3.438	3.419	3.375	3.318	3.282	3.144	3.095	3.004	2.955
	C	3.507	3.507	3.507	3.502	3.471	3.462	3.440	3.422	3.390	3.341	3.296	3.172	3.096	3.052	3.008
MCL	A	3.505	3.505	3.490	3.465	3.418	3.388	3.362	3.330	3.315	3.296	3.278	3.110	3.073	3.023	2.870
	B	3.507	3.505	3.478	3.460	3.434	3.410	3.385	3.348	3.335	3.318	3.290	3.070	3.004	2.988	2.761
	C	3.557	3.549	3.512	3.494	3.456	3.414	3.398	3.364	3.330	3.312	3.292	3.140	3.094	3.061	3.014
TR	A	3.514	3.510	3.506	3.501	3.501	3.488	3.470	3.440	3.345	3.322	3.247	3.172	3.112	3.087	3.002
	B	3.509	3.507	3.502	3.498	3.487	3.465	3.430	3.380	3.318	3.310	3.120	3.042	3.005	2.856	2.750
	C	3.502	3.501	3.501	3.500	3.492	3.480	3.450	3.422	3.370	3.312	3.226	3.178	3.080	3.004	2.856
R	A	3.505	3.503	3.503	3.490	3.487	3.458	3.432	3.418	3.374	3.312	3.270	3.211	3.172	3.050	3.002
	B	3.515	3.515	3.510	3.505	3.500	3.500	3.488	3.456	3.412	3.260	3.197	3.090	3.002	2.946	2.892
	C	3.501	3.500	3.500	3.496	3.486	3.449	3.427	3.372	3.354	3.276	3.202	3.170	3.048	3.004	2.990
BVF	A	3.513	3.511	3.508	3.504	3.500	3.489	3.480	3.435	3.398	3.320	3.240	3.168	3.090	3.046	3.002
	B	3.518	3.515	3.512	3.510	3.502	3.500	3.494	3.478	3.418	3.380	3.270	3.199	3.102	3.076	3.005
	C	3.510	3.508	3.502	3.502	3.500	3.490	3.470	3.346	3.390	3.348	3.270	3.182	3.090	3.008	2.878
	D	3.506	3.504	3.504	3.504	3.496	3.487	3.470	3.427	3.379	3.315	3.240	3.151	3.067	3.014	3.008
SCL	A	3.518	3.514	3.510	3.506	3.506	3.499	3.487	3.440	3.396	3.346	3.290	3.198	3.112	3.074	3.009
	B	3.512	3.510	3.506	3.502	3.500	3.497	3.483	3.450	3.389	3.338	3.280	3.180	3.090	2.987	2.768
	C	3.510	3.509	3.505	3.501	3.500	3.490	3.473	3.440	3.380	3.330	3.258	3.160	3.088	2.877	2.678
	D	3.508	3.506	3.500	3.500	3.500	3.488	3.464	3.432	3.380	3.320	3.241	3.148	3.096	3.002	2.876

## 2 Experimental design, materials and methods

### 2.1 Analysis of AOP

The AOP is fallen steadily with the growth of temperature [5]. At any particular temperature, the AOP of a QCA cell can be estimated by only taking the variance between highest or maximum polarization and lowest or minimum polarization and dividing the outcome by two.

(1) $$\mathrm{AOP}=\frac{\mathrm{Maximum}-\mathrm{Minimum}}{2}$$

**Fig. 1:**
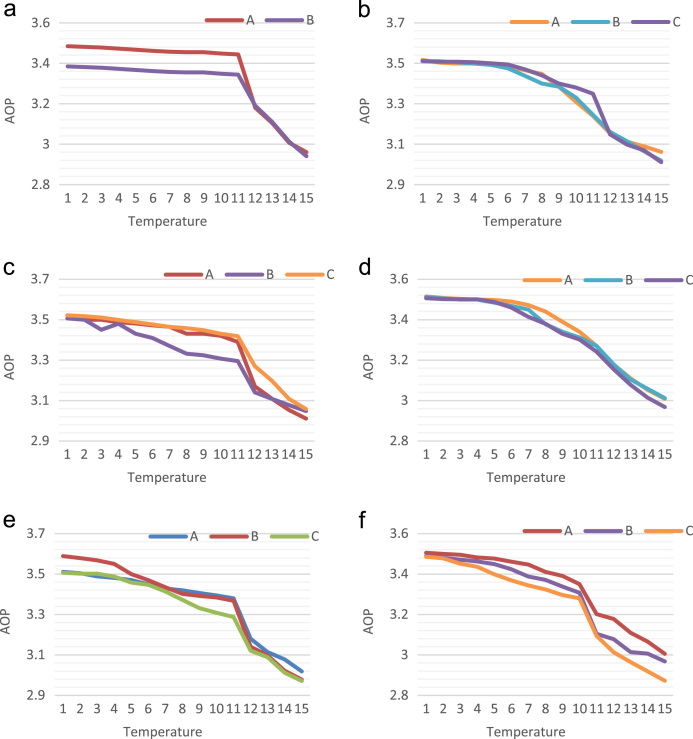

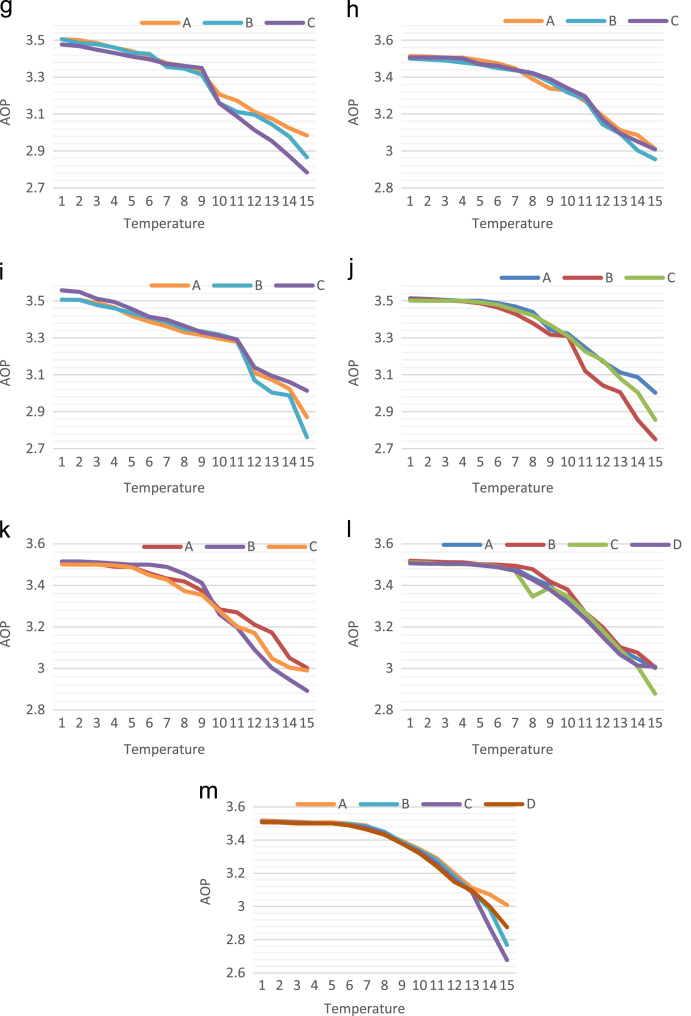
Temperature consequence on average output polarization of (a) Feynman, (b) Double Feynman, (c) Fredkin, (d) Toffoli, (e) Peres, (f) BJN, (g) URG, (h) NG, (i) MCL, (j) TR, (k) R, (l) BVF and (m) SCL circuit.

For testing the AOP, QCADesigner engine 2.0.3 has been applied with coherent vector simulation device. The succeeding default factors have been measured. The default factors are listed as: cell size=18 nm, dimension of quantum dots = 5 nm, radius of effect = 65 nm, relative permittivity = 12.90, samples number = 50,000, clock high = 9.8e^−22^ J, clock low = 3.8e^−23^ J, layer separation = 11.5 nm, convergence tolerance = 0.001, clock amplitude factor=2.00, and highest iterations for each sample = 100. The graphical depiction of AOP of various reversible circuits organized in [1], [2], [3], [4] is showed in Fig. 1.

A relative assessment of AOP by the reversible circuits is organized in Fig. 1.

## References

[1] Abdullah-Al-Shafi Md, Bahar A.N. (2017). A novel binary to grey and grey to binary code converter in majority voter-based QCA Nanocomputing. J. Comput. Theor. Nanosci..

[2] Bahar Ali Newaz, Rahman Mohammad Maksudur, Nahid Nur Mohammad, Hassan Md. Kamrul (2016). Energy dissipation data of reversible logic gates in quantum dot-cellular automata. Data Brief.

[3] Abdullah-Al-Shafi Md, Bahar A.N., Islam M.S. (2015). A quantitative approach of reversible logic gates in QCA. J. Commun. Technol. Electron. Comput. Sci..

[4] Abdullah-Al-Shafi Md (2016). Synthesis of peres and R logic circuits in nanoscopic scale. Commun. Appl. Electron..

[5] Pudi Vikramkumar, Sridharan K. (2011). Efficient design of a hybrid adder in quantum-dot cellular automata. IEEE Trans. Very Large Scale Integr. Syst..

